# Sustained Effects of CGRP Blockade on Cortical Spreading Depolarization-Induced Alterations in Facial Heat Pain Threshold, Light Aversiveness, and Locomotive Activity in the Light Environment

**DOI:** 10.3390/ijms232213807

**Published:** 2022-11-09

**Authors:** Satoshi Kitagawa, Chunhua Tang, Miyuki Unekawa, Yohei Kayama, Jin Nakahara, Mamoru Shibata

**Affiliations:** 1 Department of Neurology, Keio University School of Medicine, 35 Shinanomachi, Shinjuku-ku, Tokyo 160-8582, Japan; 2 Department of Neurology and Centre for Clinical Neuroscience, Daping Hospital, Third Military Medical University, Chongqing 400042, China; 3 Department of Neurology, Tokyo Dental College Ichikawa General Hospital, Chiba 272-8513, Japan

**Keywords:** migraine, cortical spreading depolarization, calcitonin gene-related peptide (CGRP), migraine postdrome, sensitization

## Abstract

A migraine is clinically characterized by repeated headache attacks that entail considerable disability. Many patients with migraines experience postdrome, the symptoms of which include tiredness and photophobia. Calcitonin gene-related peptide (GGRP) is critically implicated in migraine pathogenesis. Cortical spreading depolarization (CSD), the biological correlate of migraine aura, sensitizes the trigeminovascular system. In our previous study, CSD caused hypomotility in the light zone and tendency for photophobia at 72 h, at which time trigeminal sensitization had disappeared. We proposed that this CSD-induced disease state would be useful for exploring therapeutic strategies for migraine postdrome. In the present study, we observed that the CGRP receptor antagonist, olcegepant, prevented the hypomotility in the light zone and ameliorated light tolerability at 72 h after CSD induction. Moreover, olcegepant treatment significantly elevated the threshold for facial heat pain at 72 h after CSD. Our results raise the possibility that CGRP blockade may be efficacious in improving hypoactivity in the light environment by enhancing light tolerability during migraine postdrome. Moreover, our data suggest that the CGRP pathway may lower the facial heat pain threshold even in the absence of overt trigeminal sensitization, which provides an important clue to the potential mechanism whereby CGRP blockade confers migraine prophylaxis.

## 1. Introduction

Migraine is known as the second leading cause of yeas lived with disability worldwide [1]. This disabling disease is primarily characterized by recurrent moderate to severe headache attacks that are preceded and/or accompanied by transient neurological symptoms termed aura in approximately 30% of cases [2]. However, it is appreciated that patients with episodic migraines often experience disability even in the interictal (headache-free) phase [3,4,5]. In particular, approximately 80% of migraineurs have residual non-headache symptoms during the period shortly after headache attacks [6]. These residual postictal symptoms, termed postdrome, while less disabling than headache attacks, sometimes restrict daily activity, thus causing loss of productivity and well-being [6,7]. Accordingly, postdrome worsens and prolongs the suffering of those affected by migraine attacks. Nevertheless, a treatment strategy for this underrecognized condition remains unestablished. Tiredness, difficulty in concentration, and light hypersensitivity (photophobia) are among the commonly encountered postdromal symptoms [6,7]. In contemporary society, we are obliged to be exposed to light sources on many occasions by using light-emitting devices such as computer screens and mobile phones [8]. Hence, the management of photophobia is important for regaining activity and productivity after migraine attacks.

Cortical spreading depolarization (CSD) is a concentrically propagating wave of abrupt and sustained near-complete breakdown of transmembrane ion gradient and mass depolarization in the brain tissue [9]. CSD is the neurophysiological correlate of migraine aura [10,11,12,13,14,15,16]. In addition, CSD has been shown to activate the trigeminal system [17,18,19], implying that CSD may be relevant to migraine headache as well as migraine aura. Hence, CSD is widely used as a migraine model in animal studies [20].

Calcitonin gene-related peptide (CGRP) is critically implicated in migraine pathogenesis because the pharmacological interventions that block the CGRP pathway have shown to be efficacious for migraines [21]. CGRP is expressed in primary trigeminal neurons with abundant innervations of trigeminal nociceptors, especially around meningeal vessels, in the dura [22,23]. Preclinical studies show that trigeminal activation leads to CGRP release from the meningeal trigeminal fibers [24,25,26]. CGRP levels are shown to be elevated in the external jugular veins during migraine attacks [27,28]. There is ample evidence showing that CGRP induces the sensitization of nociceptors [29,30,31,32,33,34]. Upon the development of trigeminal meningeal nociceptor sensitization, normally innocuous mechanical stimulation from vascular pulsation transforms into noxious stimuli, which is ultimately perceived as throbbing pain in the brain [23].

5-HT_1B/1D_ agonists (triptans) and calcitonin gene-related peptide (CGRP) receptor antagonists (gepants) are used for acute treatment of migraine. Triptans are known to inhibit CGRP release [24,35,36]. We previously showed that CSD-induced facial hyperalgesia (surrogate marker for headache), photophobia, and hypomotility were ameliorated by administration of sumatriptan (triptan) and olcegapant (gepant) at 24 h after CSD induction in mice [37]. Furthermore, we found that CSD-subjected mice developed hypomotility and tendency for light intolerability in the absence of facial hyperalgesia at 72 h after CSD induction [38]. We presume that such delayed CSD-induced abnormalities may serve as a migraine postdrome model. In the present study, we aim to investigate the effect of the above-mentioned pharmacological interventions on the CSD-induced postdrome-like phenomena.

## 2. Results

### 2.1. Experimental Timelines

**Figure 1:**
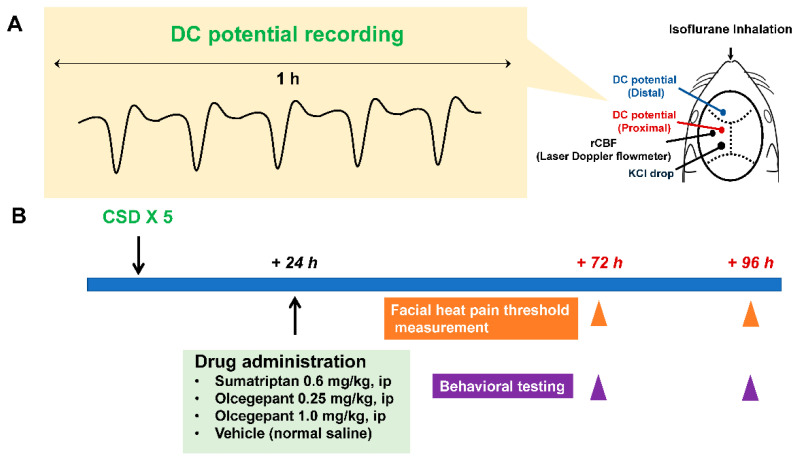
Schematic representation depicting experimental design and CSD induction. As shown in the upper left figure, CSD was induced five times within one hour (**A**). The experimental timeline of CSD induction, facial heat pain threshold temperature measurement, and behavioral testing in the present study is illustrated (**B**).

Experimental timelines for CSD induction, facial heat pain threshold temperature measurement, and behavioral assessments are depicted in Figure 1. All mice were subjected to CSD five times within one hour, and no mortality or severe morbidity was observed.

### 2.2. Temporal Profiles of Heat Pain Threshold Temperature after CSD

**Figure 2:**
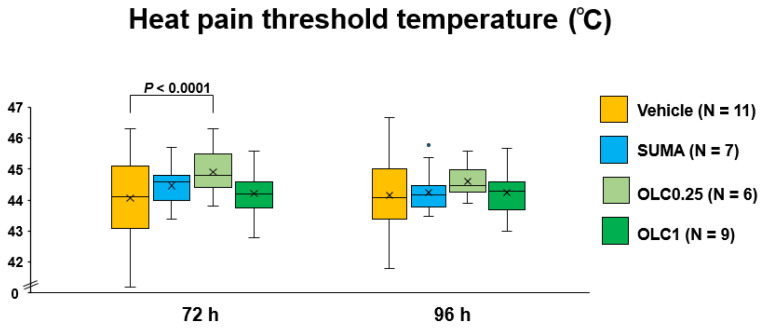
Facial heat pain threshold temperatures after CSD. The ordinate indicates facial heat pain threshold temperature (°C). Data are shown in the box-and-whisker plot. In all mice, measurements were repeated five times at each timepoint. The two-way repeated measures ANOVA were used to evaluate the effects of time and pharmacological interventions on post-CSD facial heat pain threshold temperature. Multiple comparisons were made by Sidak’s test. Vehicle: Vehicle group, SUMA: Sumatriptan group, OLC0.25: Olcegepant 0.25 mg group, OLC1: Olcegepant 1 mg group. The number of animals used are shown in parentheses.

As depicted in Figure 1, we examined the effects of pharmacological interventions with sumatriptan and olcegepant on facial heat pain threshold temperatures at 72 h and 96 h after CSD induction. The two-way ANOVA detected a significant main effect of pharmacological interventions (F_(3, 335)_ = 8.19, *p* < 0.0001), while there was no significant interaction between the pharmacological interventions and time (F_(3, 335)_ = 1.091, *p* = 0.3530). At 72 h after CSD, there was a significant difference in heat pain threshold temperature between the Vehicle group and the Olcegepant 0.25 group (44.1 ± 1.3 °C (Vehicle group) vs. 44.9 ± 0.7 °C (Olcegepant 0.25 group); mean difference: 0.84 (95% confidence interval (CI): 0.39–1.29) °C, *p* < 0.0001, Sidak’s multiple comparison test, Figure 2). At 96 h after CSD, the heat pain threshold temperature tended to be more elevated in the Olcegepant 0.25 group than in the Vehicle group (44.6 ± 0.5 °C vs. 44.2 ± 1.0 °C; mean difference: 0.44 (95% CI: 0.01–0.89) °C, *p* = 0.0539, Sidak’s multiple comparison test, Figure 2). At both timepoints, there were no significant differences in the heat pain threshold temperature among the Vehicle group, the Sumatriptan group, and the Olcegepant 1.0 group.

### 2.3. Effects of Sumatriptan and Olcegepant on Total Time Spent in the Light Zone after CSD

We examined locomotive activity in all the experimental groups at 72 h and 96 h after CSD. To assess the tolerability to light, we measured the total time spent in the light zone during the entire evaluation period for locomotion (30 min) at 72 h and 96 h after CSD.

**Figure 3:**
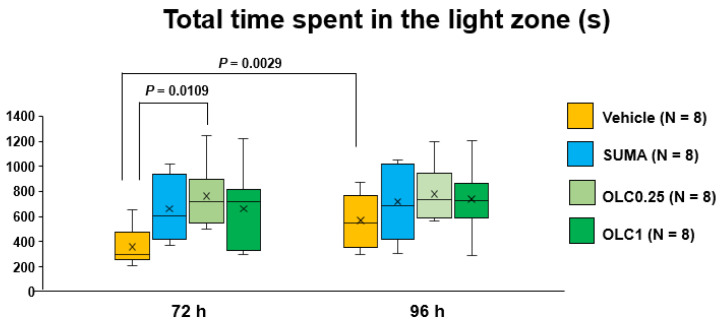
Total time spent in the light zone in each group. Data are plotted in the box-and-whisker box. The ordinate represents the sum of time spent in the light zone. Statistical analysis was performed using the two-way repeated measures ANOVA with effective matching to evaluate the effects of time and pharmacological interventions. Multiple comparisons were made by Sidak’s test. N = 8 in each group. Vehicle: Vehicle group, SUMA: Sumatriptan group, OLC0.25: Olcegepant 0.25 mg group, OLC1: Olcegepant 1 mg group.

The two-way ANOVA detected a main effect of time (F_(1, 28)_ = 10.66, *p* = 0.0027) without significant interaction with the pharmacological interventions (F_(3, 28)_ = 2.246, *p* = 0.1049). In multiple comparisons, the Vehicle group spent a significantly prolonged time in the light zone at 96 h than at 72 h after CSD (663.8 ± 259.3 s vs. 358.7 ± 150.9 s; mean difference: 211.5 (95% CI: 63.1–359.9) s, *p* = 0.0029, Sidak’s multiple comparison test, Figure 3). Moreover, there was a significant difference in time spent in the light zone at 72 h after CSD between the Vehicle group and the Olcegepant 0.25 group (358.7 ± 150.9 s (Vehicle group) vs. 764 ± 244.2 s (Olcegepant 0.25 group); mean difference: 405.8 (95% CI: 73.05–738.5) s, *p* = 0.0109, Dunnett’s multiple comparison test, Figure 3). At both timepoints, there were no differences in the total time spent in the light zone among the Vehicle group, the Sumatriptan group and the Olcegepant 1.0 group.

### 2.4. Effects of Sumatriptan and Olcegepant on Ambulatory Time and Ambulatory Distance in the Light Zone after CSD

**Figure 4:**
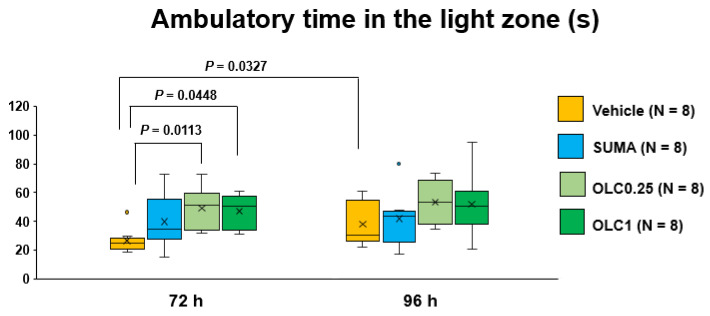
Ambulatory time in the light zone in each group. Data are plotted in the box-and-whisker box. The ordinate represents the sum of ambulatory time spent in the light zone during the evaluation period. Statistical analysis was performed using the two-way repeated measures ANOVA with effective matching to evaluate the effects of time and pharmacological interventions. Multiple comparisons were made by Sidak’s test for identical groups between 72 h and 96 h and by Dunnett’s test for different groups at the same timepoint. N = 8 in each group. Vehicle: Vehicle group, SUMA: Sumatriptan group, OLC0.25: Olcegepant 0.25 mg group, OLC1: Olcegepant 1 mg group.

As regards the effects of sumatriptan and olcegepant on ambulatory time in the light zone at 72 h and 96 h after CSD, the two-way ANOVA was found to have a significant main effect of time (F_(1, 28)_ = 6.456, *p* = 0.0169). There was no significant interaction between time and pharmacological interventions (F_(3, 28)_ = 1.115, *p* = 0.3296). In the Vehicle group, there was a significant prolongation between 72 h and 96 h after CSD (38.1 ± 15.1 s vs. 26.5 ± 8.7 s; mean difference 11.6 (95% CI: 0.7–22.5) s, *p* = 0.0327, Sidak’s multiple comparison test, Figure 4). Moreover, olcegepant of either dose increased the ambulatory time in the light zone as compared to the vehicle at 72 h after CSD (51.7 ± 19.8 s (Olcegepant 0.25 group) and 47.2 ± 11.6 s (Olcegepant 1.0 group) vs. 26.5 ± 8.7 s (Vehicle group); mean differences: 25.1 (95% CI: 4.9–45.4) s, *p* = 0.0113 (Olcegepant 0.25 group vs. Vehicle group) and 20.7 (95% CI: 0.4–40.9) s, *p* = 0.0448 (Olcegepant 1.0 group vs. Vehicle group), Dunnett’s multiple comparison test, Figure 4). No significant difference was detected between the Vehicle group and the Sumatriptan group. At 96 h after CSD, there were no significant differences in the ambulatory time in the light zone among all the experimental groups (Figure 4).

**Figure 5:**
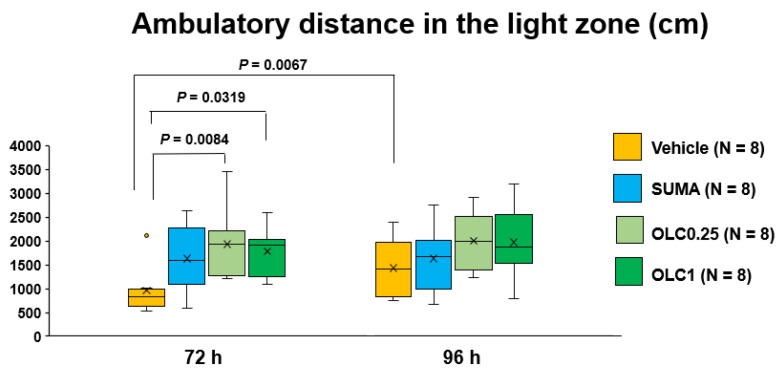
Ambulatory distance in the light zone in each group. Data are plotted in the box-and-whisker box. The ordinate shows the sum of ambulatory distance travelled in the light zone during the evaluation period. Statistical analysis was performed using the two-way repeated measures ANOVA with effective matching to evaluate the effects of time and pharmacological interventions. Multiple comparisons were made by Sidak’s test for identical groups between 72 h and 96 h and by Dunnett’s test for different groups at the same timepoint. N = 8 in each group. Vehicle: Vehicle group, SUMA: Sumatriptan group, OLC0.25: Olcegepant 0.25 mg group, OLC1: Olcegepant 1 mg group.

We also explored the effects of the pharmacological interventions on the ambulatory distance in the light zone at 72 h and 96 h after CSD. The two-way ANOVA revealed a significant main effect of time (F_(1, 28)_ = 7.709, *p* = 0.0097), while there was no significant interaction with the pharmacological interventions (F_(3, 28)_ = 2.279, *p* = 0.1012). At 72 h, both doses of olcegepant significantly extended the ambulatory distance in the light zone (1938.9 ± 732.1 cm (Olcegepant 0.25 group) cm and 1779.5 ± 494.2 cm (Olcegepant 1.0 group) vs. 957.1 ± 494.8 s (Vehicle group); mean differences: 981.8 (95% CI: 217.9–1740) cm, *p* = 0.0084 (Olcegepant 0.25 group vs. Vehicle group) and 822 (95% CI: 58.5–1586) cm, *p* = 0.0319 (Olcegepant 1.0 group vs. Vehicle group), Dunnett’s multiple comparison test, Figure 5). There was no significant difference between the Vehicle group and the Sumatriptan group. At 96 h after CSD, there were no between-group differences (Figure 5).

### 2.5. Effects of Sumatriptan and Olcegepant on Ambulatory Time and Ambulatory Distance in the Dark Zone after CSD

Next, we examined the effects of administration with sumatriptan and olcegepant on ambulatory time and ambulatory distance in the dark zone at 72 h and 96 h after CSD.

**Figure 6:**
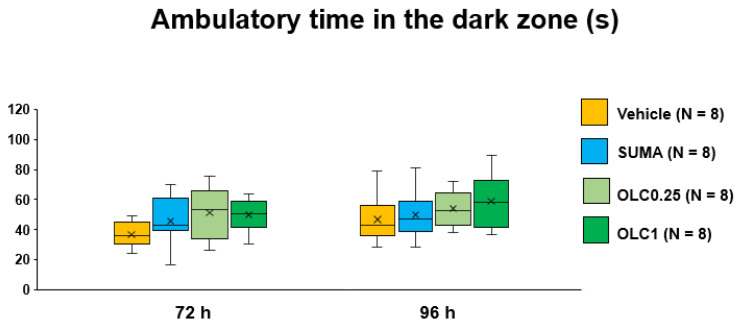
Ambulatory time in the dark zone in each group. Data are plotted in the box-and-whisker box. The ordinate represents the sum of ambulatory time spent in the dark zone during the evaluation period. Statistical analysis was performed using the two-way repeated measures ANOVA with effective matching to evaluate the effects of time and pharmacological interventions. Multiple comparisons were made by Sidak’s test for identical groups between 72 h and 96 h and by Dunnett’s test for different groups at the same timepoint. N = 8 in each group. Vehicle: Vehicle group, SUMA: Sumatriptan group, OLC0.25: Olcegepant 0.25 mg group, OLC1: Olcegepant 1 mg group.

**Figure 7:**
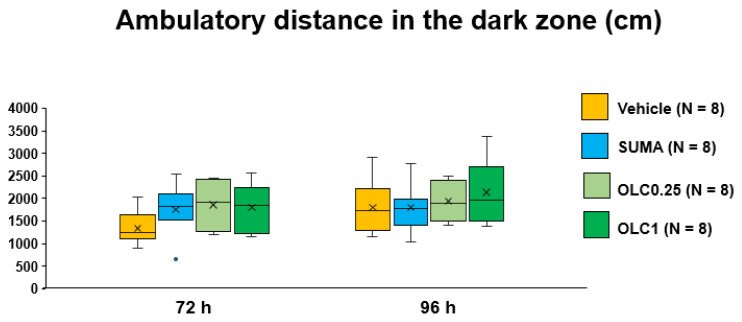
Ambulatory distance in the dark zone in each group. Data are plotted in the box-and-whisker box. The ordinate shows the sum of ambulatory distance travelled in the light zone during the evaluation period. Statistical analysis was performed using the two-way repeated measures ANOVA with effective matching to evaluate the effects of time and pharmacological interventions. Multiple comparisons were made by Sidak’s test for identical groups between 72 h and 96 h and by Dunnett’s test for different groups at the same timepoint. N = 8 in each group. Vehicle: Vehicle group, SUMA: Sumatriptan group, OLC0.25: Olcegepant 0.25 mg group, OLC1: Olcegepant 1 mg group.

The two-way ANOVA detected a significant main effect of time (F_(1, 28)_ = 9.662, *p* = 0.0043) on ambulatory time in the dark zone. No significant interaction was found between time and pharmacological interventions (F_(3, 28)_ = 0.718, *p* = 0.5496). Multiple comparisons showed that there were no between-group differences at either timepoint (Figure 6). With respect to the ambulatory distance, similar results were obtained with the two-way ANOVA, such that only a significant main effect of time was detected (F_(1, 28)_ = 13.5, *p* = 0.0010) without a significant interaction between time and pharmacological interventions (F_(3, 28)_ = 2.382, *p* = 0.0907) or between-group differences at either timepoint (Figure 7).

### 2.6. Comparison of the Average Ambulatory Speed among Experimental Groups

The average ambulatory speed was measured in each mouse to examine whether the pharmacological interventions would affect post-CSD motor function at 72 h and 96 h. The two-way ANOVA did not reveal any main effects of time (F_(1, 28)_ = 0.3101, *p* = 0.5820) or pharmacological interactions (F_(3, 28)_ = 1.104, *p* = 0.3638). Significant between-group differences were not detected in average ambulatory speed at 48 h (Vehicle group: 58.7 ± 4.2 cm/s, Sumatriptan group: 61.7 ± 3.7 cm/s, Olcegepant 0.25 group: 57.9 ± 3.3 cm/s and Olcegepant 1.0 group: 58.3 ± 3.9 cm/s, Dunnett’s multiple comparison test) or at 72 h (Vehicle group: 60.1 ± 3.8 cm/s, Sumatriptan group: 60.7 ± 5.6 cm/s, Olcegepant 0.25 group: 57.7 ± 6.3 cm/s and Olcegepant 1.0 group: 59.8 ± 3.8 cm/s, Dunnett’s multiple comparison test).

## 3. Discussion

The present study showed that CGRP receptor blockade at 24 h after CSD, when facial hyperalgesia was present, suppressed CGRP-induced postdrome-like phenomena, such as light aversion and hypomotility in the light zone. This finding highlights the importance of early inhibition of the CGRP pathway in the amelioration of postdrome as well as ictal symptoms in migraine. Additionally, it was revealed that the CGRP receptor blockade rendered mice as less sensitive to heat pain in the trigeminal territory at 72 h after CSD. Hence, our observation implies that the CGRP pathway may play an important role in determining the threshold for heat pain development in the trigeminal territory. CSD is known to sensitize the trigeminal system at the first and second order levels [17,18]. Strassman et al. [39] recently reported that CSD-induced activation of C- and A-meningeal nociceptors was mediated by CGRP. Cyclicity of headache attacks is a salient feature of migraine [2]. Heat pain thresholds are shown to be lower in patients with migraines at the forehead during the interictal (headache-free) period than in normal controls [40]. Moreover, there were positive correlations between heat pain thresholds and number of hours until the next migraine, which suggests that the magnitude of sensitization may be most elevated immediately before the next bout. The spinal trigeminal nucleus is shown to increase the activation level to trigeminal nociceptive stimulation over the interictal period toward the subsequent attack [41]. These observations indicate that the trigeminal sensitization fluctuates during the migraine interictal period and has thresholds for evoking the next headache attack in migraineurs. The sustained effect of olcegepant on facial heat pain threshold in our study seems to support the tenet that CGRP blockade exerts a migraine prophylactic effect by attenuating trigeminal sensitization.

Patients with migraine often report photophobia during postdrome as well as during the ictal phase [6,7]. Furthermore, enhanced activation was observed during postdrome in the visual cortex by functional MRI analysis [42]. In the present study, olcegepant treatment resulted in increased tolerability to light at 72 h after CSD. Furthermore, there was a tendency for the sumatriptan-treated mice to spend more time in the light zone as compared to the vehicle-treated mice (Figure 3). This observation also lends support for the role of CGRP in the regulation of photosensitivity, because sumatriptan is shown to inhibit CGRP release [24,35,36]. In the present study, olcegepant at the dose of 0.25 mg/kg exerted consistently favorable actions on the behavioral parameters as compared to sumatriptan. This suggests that direct CGRP blockade may be a better therapeutic option for amelioration of postictal symptoms than 5-HT_1B/1D_ blockade. Our data demonstrated that CSD caused hypomotility at 72 h only in the light zone. Concomitantly, olcegepant improved the CSD-induced hypomotility in the light zone with respect to ambulatory time and ambulatory distance. This hypomotility cannot be explained by motor dysfunction because average ambulatory speed was normal. Considering that there was no definite hypomotility in the dark zone at the same timepoint, we reasoned that light aversion played an important role here. The ability of olcegepant to improve mobility in the light zone seems clinically important. Decreased physical activity has been reported in migraineurs during the interictal phase not limited to the postdromal phase, and physical impairment is now regarded as a clinically important patient-reported outcome [43]. Our data indicate that CGRP inhibition during the ictal phase may prevent photophobia and hypomotility in the light environment during postdrome, which is likely to contribute to improving the quality of life and productivity at work.

There are several limitations to the present study. First, we adopted a CSD-based model, which is suitable for studying the disease state associated with migraine with aura. Because the majority of migraineurs have migraine without aura, caution should be taken in extrapolating our observations to patients with migraine. Second, we used only male mice in the present experiments with a view to avoiding the potential effects of the menstrual cycle on the elicitability of CSD [44]. However, migraines are three times more common in women than in men, and sex steroids and prolactin are likely to be implicated in migraine pathophysiology [45,46]. Moreover, there seem to be sex differences in response to triptans [47]. Hence, the data obtained only from male mice in the present study may not be applicable to women with migraines. Third, it is uncertain how the efficacy of olcegepant witnessed in the present study should be correlated with its clinical effectiveness on actual migraine. The optimal dose of olcegepant for murine translational research of migraine remains established. As we found that the effects of olcegepant on trigeminal sensitization, light aversion, and physical activity in the light environment differed depending on its dose, it may be imperative to determine its optimal dose by using further dose-titration in future studies. Moreover, it is obscure as to why single administration of olcegepant could exert biological effects at 48 h post-dose in the present study. Judging from its half-life of 2.5 h [48], the CGRP receptor-blocking effect of olcegepant per se should have worn off. It seems more likely that the inhibition of the CGRP pathway during the acute period in the presence of trigeminal sensitization would alter the trajectory of trigeminal sensitization and prevent the emergence of delayed photophobia and hypomotility in the light environment.

Despite these limitations, the present study provides an important insight into the mechanism whereby CGRP blockade confers migraine prophylaxis. Moreover, our data raise the possibility that ictal CGRP receptor blockade may ameliorate migraine postdrome, thereby enhancing the quality of life and productivity at work, thus underscoring the importance of pre-emptive anti-CGRP therapy in migraine attacks.

## 4. Materials and Methods

### 4.1. Animals

This study was approved by the Laboratory Animal Care and Use Committee of Keio University (No. 14084). All experimental procedures were performed in accordance with the university-approved protocols and EC Directive 86/609/EEC for animal experiments. Male C57BL/6 mice aged 8–10 weeks were purchased from CLEA Japan Inc. (Fujinomiya, Japan). A total of 65 mice were used for the present study. They were housed in an ambient specific-pathogen-free condition with a 12-h light/dark cycle and given food and water ad libitum.

### 4.2. CSD Induction

Under isoflurane anesthesia (1–2%), the mouse head was fixed in a stereotaxic apparatus. Systolic blood pressure and heart rate were monitored at the tail artery with a non-invasive blood pressure monitoring machine (MK-2000ST; Muromachi Kikai Co., Ltd., Tokyo, Japan). Rectal temperature was maintained at approximately 37 °C using a heating-pad and thermocontroller (BWT-100; Bioresearch Center Co., Ltd., Nagoya, Japan).

The detailed procedures of the electrophysiological recording were described previously [49]. A midline incision of the scalp was made to expose the skull. Three small holes were drilled in the skull over the left hemisphere with the underlying dura intact. The posterior hole, centered at the coordinates of 5 mm posterior and 2 mm lateral to the bregma, was made for the KCl stimulation. The parietal hole (2 mm lateral and 2 mm caudal to the bregma) and the frontal hole (2 mm lateral and 2 mm rostral to the bregma) were made for the installation of the recording electrodes. Two Ag/AgCl DC electrodes (tip diameter = 200 μm, EEG-5002Ag; Bioresearch Center Co., Ltd., Nagoya, Japan) were placed on the dura at the parietal (proximal) and frontal (distal) holes, respectively, with a differential head stage and differential extracellular amplifier (Model 4002 and EX1; Dagan Co., Minneapolis, MN, USA). In the proximity of the parietal hole (4 mm lateral and 2 mm posterior to the bregma), the probe (BF52; Advance Co., Ltd., Tokyo, Japan) of a laser Doppler flowmeter (LDF; ALF 21, Advance Co., Ltd.) was installed on the intact skull to monitor the regional cerebral blood flow (rCBF). Continuous recordings of the DC potential and rCBF were stored on a multi-channel recorder (PowerLab 8/30; ADInstruments, Ltd., Sydney, Australia), and LabChart software (ADInstruments, Ltd.) was used for off-line analysis as reported previously. CSD was induced by stimulation with KCl solution (1.0 mol/L, 5 μL) onto the dura. The appearance of CSD was confirmed by the demonstration of a distinct DC potential deflection, typical fluctuation of rCBF, propagation to the distal portion, and the suppression of electroencephalography (Figure 1B). The CSD induction was performed five times over 20–30 min. After the surgery, all electrodes were removed, and the craniotomies were closed off with dental cement. We confirmed that intraoperative hemodynamic parameters (heart rate (bpm) and systolic blood pressure (mmHg)) and pre-operative body weight (g)) were within physiological ranges. We did not administer any antibiotics after surgery.

### 4.3. Drug Administration

The following administration with drugs or vehicle was carried out at 24 h after CSD induction (Figure 1A). Sumatriptan succinate (Tokyo Chemical Industry Co., Ltd., Tokyo, Japan), dissolved in normal saline, was intraperitoneally administered at a dose of 0.6 mg/kg using a 26-gauge needle. The amount of diluent was 10 µL/g. Olcegepant (MedChemExpress, Mammoth Junction, NJ, USA), initially dissolved in dimethylsufloxide at 0.025 mg/µL, was 250-fold diluted in normal saline. Intraperitoneal injections at a dose of either 1.0 mg/kg or 0.25 mg/kg were carried out through a 26-gauge needle. We used vehicle-treated mice as controls, in which normal saline was intraperitoneally administered using a 26-gauged needle. We designated experimental groups as follows. Those given with the vehicle at 24 h after the CSD induction were referred to as the Vehicle group. The other experimental groups were subjected to a pharmacological intervention at 24 h after CSD. They were designated in accordance with the administered agent and dose: the Sumatriptan, Olcegepant 0.25, and Olcegepant 1.0 groups.

### 4.4. Facial Heat Pain Threshold Temperature Measurement

The detailed protocol for heat pain threshold temperature was described previously [50]. Briefly, after acclimation to an experimental apparatus that restricted body mobility, except for head movement, a pair of Peltier module bars with surface temperature regulated between 36 °C and 56 °C was applied to the face bilaterally. The bar surface temperature gradually increased from 36 °C by 1 °C/4 s until face withdrawal. Mouse behaviors were captured employing a video recorder (Panasonic, Kadoma, Japan). The video was analyzed by an examiner that was blind to the identity of the animals. The lowest temperature at which a mouse turned the head away from the bars was recorded as the heat pain threshold temperature. In each session, measurements of the threshold temperature were recorded in pentaplicate. In our previous study, we reported that the baseline heat pain threshold temperatures did not differ among the experimental groups used for the present study [37].

### 4.5. Behavioral Analysis in the Light and Dark Zones

Mouse behaviors were continuously monitored in an open field chamber (27 cm wide × 27 cm deep × 20.3 cm high) with three sets of 16-beam infrared arrays (two sets of perpendicular beams crossed at a height of 1.0 cm to detect mouse location and locomotion, and the third beam crossed the width of the chamber at a height of 7.3 cm to detect vertical activity; Med Associates, Fairfax, VT, USA). The testing chamber was placed inside a windowless cabinet. The testing field was equally compartmented in light and dark zones by a dark insert (Med Associates). Mice could move freely between the two zones through an orifice (5.2 cm × 6.8 cm) in the dark insert. The light intensity measured at a height of 2 cm in the light zone was 540 lx, whereas the intensities measured in the dark zone were 380 lx immediately inside the orifice, 20 lx at the center, and 5 lx at the corners. Mouse activity was analyzed using a computer equipped with Activity Monitor v6.02 (Med Associates).

On the day mice were taken from the animal facility, they were allowed to acclimatize to the test chamber for 10 min with the overhead lighting off, then for 30 min with the lighting on. This acclimation process was repeated on the subsequent two days at an interval of 24 h. Previous studies revealed that daily repeated testing is possible up to three to four times a week without affecting the exploratory behavioural pattern [51,52]. On the day of the CSD or sham operation, the acclimation was carried out prior to the operation. At 24 h after CSD or sham operation, mice were intraperitoneally administered with either an active drug or vehicle (Figure 1A). Subsequently, they were put into the testing chamber with the lighting off for 10 min, and their locomotive activity was recorded for the following 30 min with the lighting on. For all mice examined, behavioural testing was carried out between 10:00 and 18:00 in a quiet room.

### 4.6. Evaluation of Mouse Locomotion

The effects of drug administration on mouse locomotion in both zones were evaluated using several parameters relevant to movement. In this experiment, when mice moved out of a 6.35 cm × 6.35 cm square box around them within 0.5 s, their movements were defined as ambulatory. We designated the total time in ambulatory movement status as ambulatory time. Ambulatory distance (cm) was defined as the total distance travelled during the ambulatory movement status. Ambulatory average velocity (cm/s) was calculated as ambulatory distance divided by ambulatory time, which was defined as the time spent in the ambulatory movement status. In the present study, the ambulatory average velocity was used as an index to evaluate individual mouse motor function. Our preliminary experiments revealed that the standard deviation of the whole time spent in the light zone of untreated control mice was 120–150 s. With the type I error rate and power being 5% and 0.80, respectively, if we were to detect a 200-s difference, the sample size required was calculated as 7–10 subjects in each group. In our previous article, it was verified that the baseline values of these locomotive parameters did not differ among the experimental groups used for the present study [37].

### 4.7. Statistical Analyses

All numerical data are expressed as the mean ± SD. The two-way repeated-measures ANOVA was used to evaluate the effects of pharmacological interventions on threshold temperatures for facial heat pain and locomotive parameters at 48 h and 72 h after CSD, followed by Dunnett’s or Sidak’s multiple comparison test. Mean differences were indicated with a 95% CI. In multiple comparisons, *p* values were adjusted for multiplicity. *p* < 0.05 was considered as statistically significant. Data were analyzed using GraphPad Prism 8 software (GraphPad Software, San Diego, CA, USA).

## Data Availability

All data generated and/or analyzed during this study are included in this published article.
